# Proposal to Employ Heated Air in Hydrophobia

**Published:** 1828-06

**Authors:** 


### HYDROPHOBIA.

### *Proposal to employ heated Air in Hydrophobia.*

NEARLY all the remedies with which we are acquainted have originated from some accident, which an observer has taken advantage of, and, by a reflective operation of the mind, has perceived to be applicable, if its effects were moderated, to the cure or alleviation of some disease or disorder of the human body. This method of reasoning, and its consequent application, is and has been productive of much good, and forms a rational ground of medical practice, even if pursued without any connexion with anatomy, physiology, or any other branch of the science of physic. But a fanciful and sort of guess application of remedies must necessarily, in general, be attended with so little benefit, and make an inroad for so much mischief, that I feel the full force of venturing the proposal of one upon such basis, and can easily foresee the ridicule which may arise from its failure. There is, however, in the instance I am contemplating such a freedom from evil in its consequences, and the case in which I propose it is so totally out of the reach of the present aid of medicine, that it becomes in its result at most an error in fancy, while it will, if successful, be a means of preserving the lives of those who are decidedly out of the reach of medical aid. It would, therefore, be an inhuman error to hold back the present proposed remedy, merely because, in advancing it, there is only a glimpse of hope, instead of a sanguine expectation.

There is a disease which is manifestly communicated by the introduction of a powerful poison into the system. When this poison has given rise to a certain phenomenon, it is invariably productive of death. This disease, like most others, has its name from its prominent symptom, which in this instance is a dread of water, (hence the term Hydrophobia.) Every remedy which has been employed here, and I believe nearly all the articles of our long list of materia medica have been essayed, has deceived the flattering hope of the unfortunate, who has been driven to a treacherous repose upon their power. In these cases, anatomical investigation affords but little light, since the exposures of the scalpel display scarcely any two cases alike; and, although traces of inflammation are discoverable, I believe, in all the cases examined, yet its seat is so uncertain, and its indications sometimes so obscure, and, when rightly guessed at, customary remedies prove so inert as to set at defiance the usual advantages of scientific procedure in common circumstances. Pathology of the disease there is none, and physiology affords not even data to explain the phenomenon which gives rise to its name. It may be urged that, if this should be explained, the disease would be understood; but I conceive this is a mistaken notion; for, although the mental emotion of fear, as connected with water or any other undulating body, is the most distressing symptom to the sufferer, yet it would not appear that this dread is the cause of death. *On the contrary, this feeling subsides, I believe, as dissolution approaches.*

Without encumbering the subject further with crude observations to no useful end, it is my wish to propose, through you, to the public, that future cases of this disease may be submitted to the full influence of highly heated air, so as to produce from the skin and lungs a copious exhalation.

This treatment affords to my mind a hope of success, because, in all the more virulent and manifestly contagious diseases, as plague, small-pox, measles, and scarlatina, there is an effort to throw off the poison by cuticular action; and, in the plague, those who get bubo and cuticular disease early do for the most part recover, and, in some instances, *spontaneous sweating has appeared to carry off the disease.*

The abuse of language is often the origin of error, but the origin of words is always in absolute truth, because it is merely the absolute and imperious application of a term to denote a fixed fact; and therefore, in attaining to the precise and exact meaning of a term in its original import, we must always acquire a knowledge of the determinate fact to which it was originally fixed as a denotement.

In the histories of nations, it appears well established that the arts and sciences rise, flourish, and decay, in a mutual conformity; and hence, where the lasting monuments of the former show the perfection to which they have been cultivated, it is at once known that the sister sciences cannot have been neglected, and the degree of excellence in the former is plainly indicative of the eminence reached by the latter. Hence the lasting monuments in art of the ancients are the powerful witnesses of their superiority in science.

Now, our terms of science, more especially of medicine, are derived from the same source as the admired remains of ancient sculpture; and the knowledge of medicine attained to by those people (the ancient Greeks,) was, in all probability, (whatever the moderns may think to the contrary,) equal to their success in the arts.

There is, derived from the Greek language, a word now rarely used by the profession, which lexicographers say means *expeller of poisons*. This word is *Alexipharmic,* and all the drugs comprehended under that term have the property of producing sweat. There would appear, therefore, some reason to believe that the ancients had a knowledge of the means of expelling poisons, and, if we have a correct application of their term to the same class of medicines which they used, it must have been by sweating; and hence an argument is raised upon their superior knowledge to the use of a sudatorium in the cure of hydrophobia.*

But not to trust to authority for what argument should enforce, it may be worth the time to inquire what would be the dictates of nature (that is the disease) to remedy this fatal and distressing evil.

The natural and evident cause of this disease is the introduction of a powerful poison into the system: the natural and evidently rational method of cure must be the expulsion of that poison, unless some medicine were known (and there is none) which possessed the property of rendering innoxious that virus. The expulsion of a poison mixed with the blood cannot, like poisons in the stomach or bowels, be removed by emetics or aperients, but must be cast off by the excretory pores of the blood-vessel system; so far the sudatorium is, in

* The name of the herb Contrayerva (or counter-poison) must have originated in its actual or supposed power, and this medicine also is a sudorific; as is also the plant Serpentaria, supposed to be efficacious against the bite of serpents.

the simplicity of genuine philosophy, clearly indicated as the first point of external cure.

To moderate the leading symptom of this disease, the dread of water, nothing appears more rational than to induce the powerful counter-desire thirst; and this the sudatorium, by its abstracting the fluids of the body, will tend powerfully to produce: so far the heated air would seem clearly indicated by the dictates of the disease.

I have before stated that there exists no pathology of the disease which is deserving of attention, and the leading phenomenon of the disease has not an explanation even plausible; but it does probably depend upon that concomitant morbid irritability of the body which is so peculiar to this kind of poisoning. There is, perhaps, no means of relieving an undue irritability of body, not dependent upon actual debility, so efficacious as the sudatorium: and hence it would appear also indicated by the nature of the disease itself.

Even if the disease be inflammatory, although the traces of inflammation are, I believe, scarcely sufficiently marked to account for death, the sudatorium would be highly useful, and strongly indicated, inasmuch as it is a powerful antiphlogistic remedy, especially in erratic disorder of the inflammatory kind.

In a state where impressions so trifling as, in the ordinary circumstances of life, would be unheeded, do excite so powerfully the animal frame, a rarer medium, as less powerful in conveying impressions, would be enforced by the salutary dictates of nature, in the same way as she seeks cooler air in fever, and warmth in debility. And hence it is probable that the sudatorium would not only be useful, but perhaps the most pleasant application to the feelings of the distressed in the disease.

These, sir, are the circumstances which influence me in hoping benefit from this practice; and I beg, through you, to submit them to the consideration of the profession at large, that they, with whom they may weigh powerfully as they do with me, may put the opinion to the test. The life of a private individual may pass without affording a case in point for experiment, so that to wait the experience of myself upon a subject so important to my fellows, would neither be justifiable nor humane; and this proposed remedy may as well be fairly and fully tried, as to suffer the miserable subjects of a hitherto universally fatal disease to lie, as they do, curious exhibitions in our hospitals for an unerring prognosis of death. It appears to me that the sweating should be long, frequent, and indeed carried to an extreme point. Z.

